# Disruption of the EZH2/miRNA/β-catenin signaling suppresses aerobic glycolysis in glioma

**DOI:** 10.18632/oncotarget.10370

**Published:** 2016-07-01

**Authors:** Yingyi Wang, Min Wang, Wenjin Wei, Dongfeng Han, Xincheng Chen, Qi Hu, Tianfu Yu, Ning Liu, Yongping You, Junxia Zhang

**Affiliations:** ^1^ Department of Neurosurgery, The First Affiliated Hospital of Nanjing Medical University, Nanjing, China; ^2^ Department of Radiology, The First Affiliated Hospital of Nanjing Medical University, Nanjing, China

**Keywords:** EZH2, miRNA, β-catenin, aerobic glycolysis, glioma

## Abstract

EZH2 is up-regulated in various cancer types, implicating its role in tumorigenesis. Our recent data have shown that repression of EZH2 inhibited glioma growth by inhibition β-catenin signaling. Here, we identified several miRNAs that were repressed by EZH2, which in turn regulate β-catenin expression by its 3′UTR, such as miR-1224-3p, miR-328 and miR-214. Further, EZH2 silenced miR-328 expression by binding to miR-328 promoter and promoting methylation of miR-328 promoter. Finally, miR-328 largely abrogated EZH2 effects on β-catenin expression and glucose metabolism in glioma cells. Taken together, we propose a model for a coordinated EZH2-β-catenin oncoprotein axis, and epigenetic link between histone modification and DNA methylation, mediated by EZH2-scilenced miRNAs.

## INTRODUCTION

The reprogramming in energy metabolism is listed as one of the ten hallmarks of cancer [1]. The Warburg effect (aerobic glycolysis), is critical for tumor cell proliferation [2]. PKM2 and IDH1 are identified as key regulators for aerobic glycolysis in gliomas [3, 4]. However, few studies on glucose metabolism in glioma cells have been reported.

EZH2, the core components of PRC2 functions as a histone methyltransferase for the trimethylation of histone 3 on lysine 27 (H3K27me3), and also recruits DNA methyltransferases to their target promoters, thereby epigenetically silencing these genes [5, 6]. Therefore, DNA methylation and histone methylation are key steps involved in EZH2 mediated epigenetic silencing. EZH2 has been to play an important role in a variety of different cancers [7]. Our recent data have shown that increased EZH2 expression was associated with tumor grade and short overall survival in gliomas [8]. Repression of EZH2 induced cell cycle arrest and inhibited tumor growth *in vivo*, by inhibition β-catenin signaling. However, further mechanisms of EZH2 in gliomagenesis remain poorly understood.

In this study, we introduced miRNAs as the key mediator between EZH2 and β-catenin signaling in glioma aerobic glycolysis. We identified several miRNAs that are repressed by EZH2, which in turn regulate β-catenin expression by its 3′UTR. And EZH2 induced histone modification and DNA methylation of miR-328. Further, miR-328 was crucial for EZH2/β-catenin signaling in glucose metabolism. To our knowledge, these data indicate for the first time that EZH2/miR-328/β-catenin signaling could be potential therapeutic targets for glioma intervention.

## RESULTS

### EZH2 activity inhibition suppresses aerobic glycolysis in glioma cells

**Figure 1:**
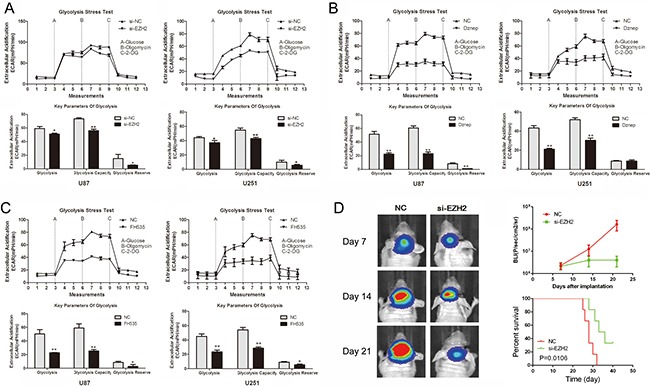
EZH2 promotes glioma cell aerobic glycolysis (**A**–**C**) ECAR was measured by the Glycolysis Stress test in U87 and U251 glioma cell lines after the cells were treated with si-EZH2, Dznep, FH535, respectively. The glycolysis under basal conditions, the glycolytic capacity and the glycolytic reserve were analyzed according to the methods. *P < 0.05, **P < 0.01. Results are representative of at least three independent experiments. (**D**) U87 cells pretreated with lentivirus containing a luciferase reporter were implanted into the brains of nude mice, and tumor formation was assessed by bioluminescence imaging. Changes in bioluminescent signal were detected at day 7, 14, and 21 after implantation. Overall survival of nude mice was determined by Kaplan–Meier survival curves and log-rank test was used to assess the statistical significance of the differences.

To explore the role of EZH2 in glioma cell glucose metabolism, glycolysis stress test was employed. Si-EZH2 transfected cells exhibited lower levels of the extracellular acidification rate (ECAR) after treatment with glucose or oligomycin compared with the negative control (Figure 1A). The glycolysis under basal conditions, the glycolytic capacity and the glycolytic reserve were both inhibited when EZH2 was decreased in U87 and U251 glioma cells. Also, the EZH2 inhibitor, Dznep, significantly inhibited glucose consumption, consistent with a role for EZH2 in extracellular acidification (Figure 1B). Our previous data showed that EZH2 knockdown triggered a reduction of β-catenin expression both in the mRNA and protein levels. Thus, we detected whether β-catenin regulate glucose metabolism in gliomas. The inhibition of β-catenin activity by FH535 decreased the level of the glycolysis under basal conditions, the glycolytic capacity and the glycolytic reserve (Figure 1C). Overall, EZH2 promotes aerobic glycolysis in glioma cells.

To further evaluate the effects of EZH2 on tumor growth *in vivo*, we established intracranial xenograft tumors in nude mice. U87 cells were pretreated with a lentivirus containing a luciferase reporter. As shown in Figure 1D, when EZH2 was inhibited, the intracranial tumor significantly decreased compared with the corresponding control group. Compared with the control group, si-EZH2-treated group showed prolonged survival until the end of the observation.

### Identification of EZH2-targeting miRNAs

**Figure 2:**
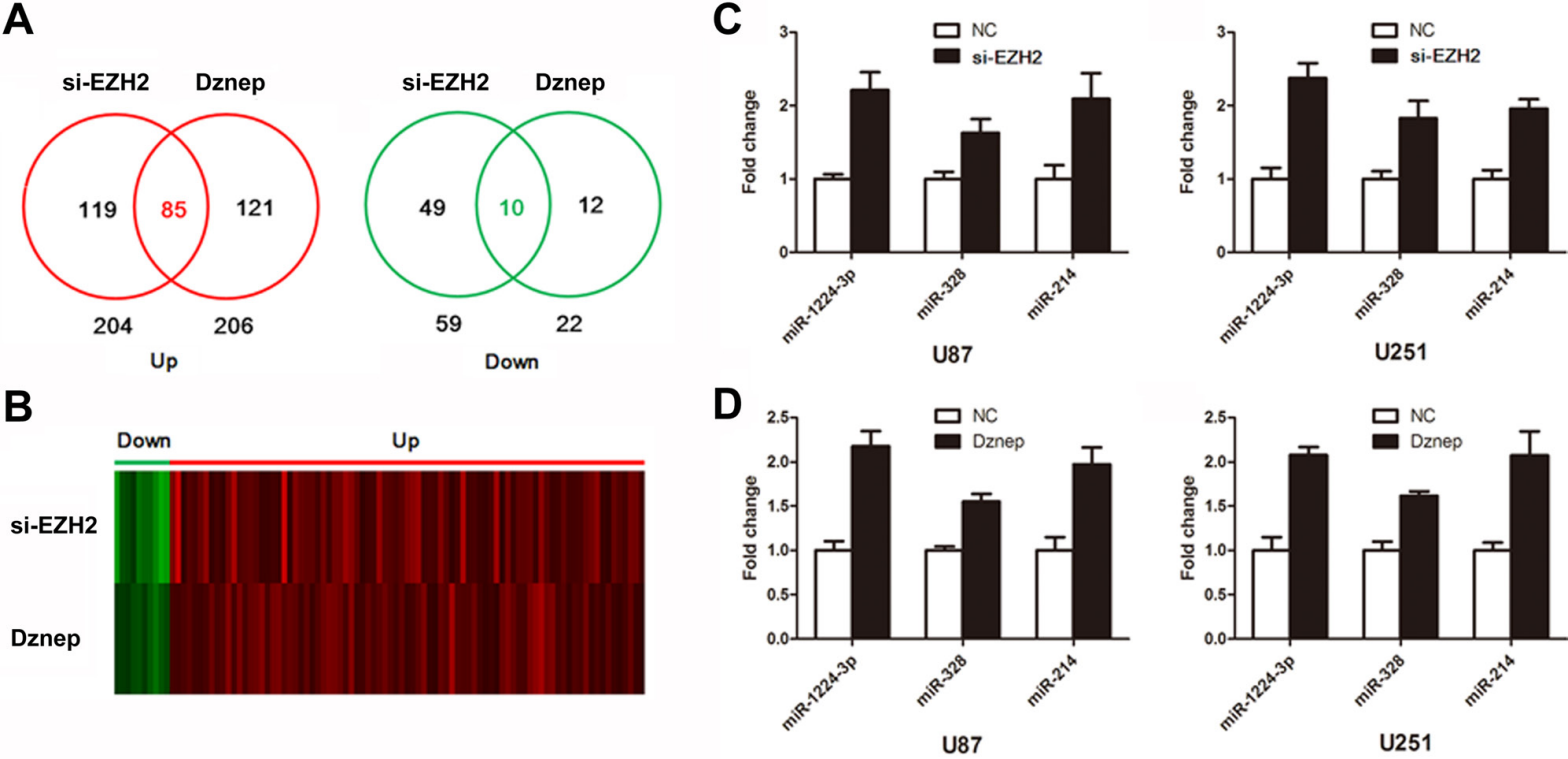
Identification of EZH2-targeting miRNAs (**A** and **B**) Venn diagram and heat map show 85 upregulated miRNAs and 10 downregulated miRNAs in glioma cells treated by si-EZH2 and Dznep. (**C** and **D**) The expression of miR-1224-3p, miR-328 and miR-214 were determined by real time PCR following treatment of si-EZH2 and Dznep in U87 and U251 cells. Results are representative of at least three independent experiments.

To identify miRNAs regulated by EZH2 globally, we inhibited EZH2 by siRNA and Dznep in U87 glioma cells and monitored miRNA expression with miRNA array. We primarily observed that 119 miRNAs were upregulated and in 49 miRNAs were downregulated in si-EZH2 transfected cells (log2|Fold change| ≥ 0.6, *P* < 0.05). And 121 miRNAs were upregulated and in 12 miRNAs were downregulated in Dznep-treated cells. Thus we identified 85 miRNAs as EZH2-targets which were both upregulated by si-EZH2 and Dznep (Figure 2A and 2B).

We hypothesized that β-catenin may in general be regulated by the EZH2-targeting miRNAs. To test this hypothesis, we used miRNA target analysis to predict whether these miRNAs bind to the 3′UTR of β-catenin. Of 85 miRNAs, 10 miRNAs were identified as candidates to target β-catenin (Table 1). Further, real time PCR showed that si-EZH2 and Dznep treatment triggered a significant reduction of miR-1224-3p, miR-328 and miR-214 in glioma cells (Figure 2C). These data suggested that miR-1224-3p, miR-328 and miR-214 maybe involved in EZH2/miRNAs/β-catenin signaling.

**Table 1:** 10 EZH2-silenced miRNAs predicted to target β-catenin in glioma

miRNA	si-EZH2			Dznep	
	log2 (Ratio)	*P*		log2 (Ratio)	*P*
miR-1224-3p	1.311058	0.000001		1.124632	0.000001
miR-574-3p	0.971511	0.000022		1.253722	0.000002
miR-214	1.562725	0.000154		1.887257	0.000001
miR-92a-1-5p	2.338155	0.000302		0.780241	0.001192
miR-328	0.664034	0.001385		0.638155	0.001546
miR-634	1.25075	0.001698		1.534439	0.000001
miR-532-3p	0.704332	0.00178		1.511351	0.000031
miR-483-3p	0.744229	0.002564		1.409354	0.000631
miR-1225-3p	0.833631	0.019461		0.822286	0.000007
miR-574-5p	1.121877	0.02296		1.177004	0.000008

### β-catenin is a target for miR-328, miR-214 and miR-1224-3p

**Figure 3:**
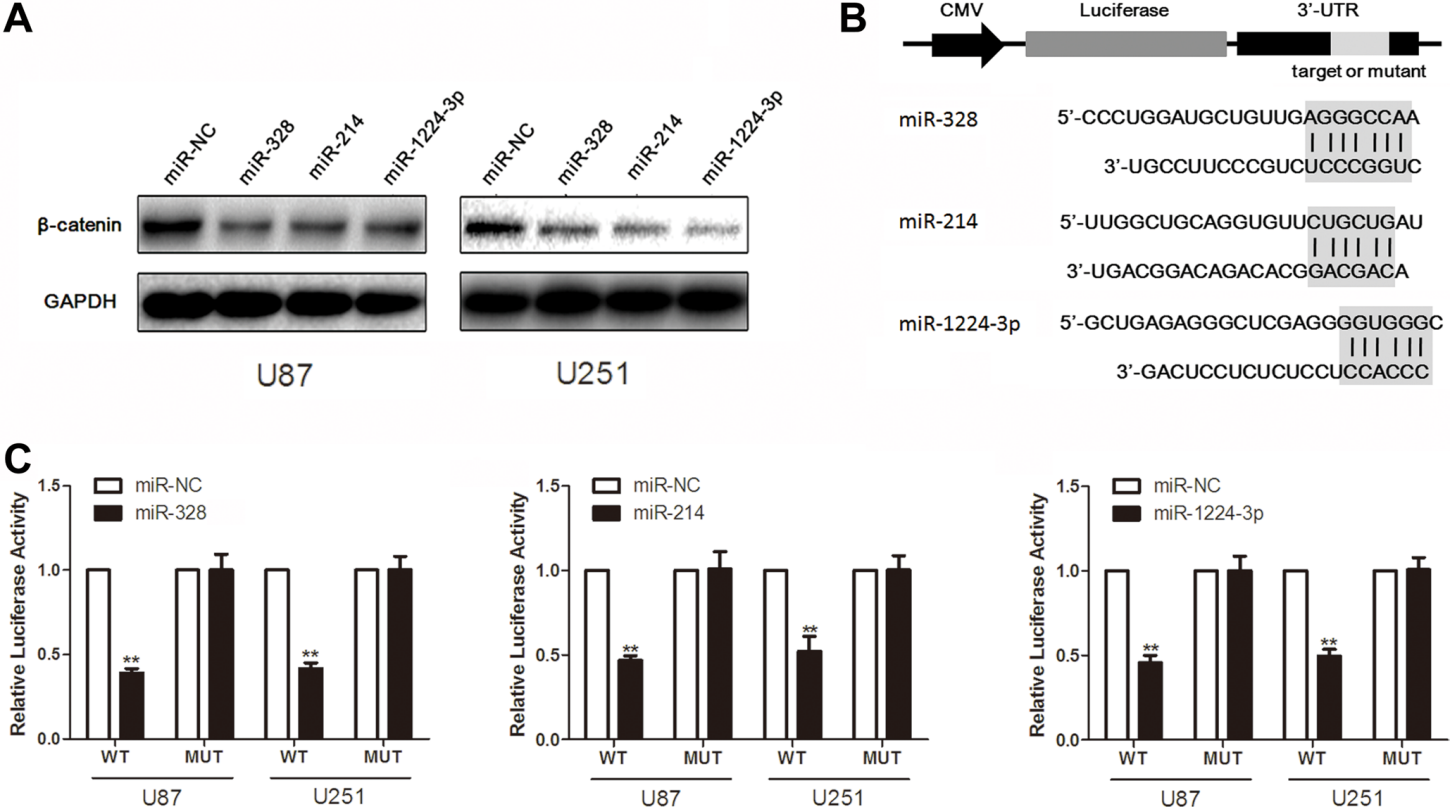
MiR-328, miR-214 and miR-1224-3p target β-catenin (**A**) Western blot analysis of lysates from cells transfected by miR-328, miR-214 or miR-1224-3p probed with β-catenin antibody. GAPDH was served as the loading control. (**B**) Schematic representation of the putative binding sites in β-catenin mRNAs 3′UTR for miR-328, miR-214 and miR-1224-3p. (**C**) pGL3-WT-β-catenin-3′UTR-Luc and pGL3-MUT-β-catenin-3′UTR-Luc reporters were transfected into glioma cells treated by miR-328, miR-214 or miR-1224-3p. Luciferase activity was determined 48 h after transfection. The ratio of normalized sensor to control luciferase activity is shown. Error bars represent standard deviation and were obtained from three independent experiments.

Western blot analysis showed that β-catenin expression was down-regulated in glioma cells with overexpression of miR-328, miR-214 and miR-1224-3p, compared to the cell treated with scrambled oligonucleotide (Figure 3A). Moreover, we created pGL3-WT-β-catenin-3′UTR and pGL3-MUT-β-catenin-3′UTR plasmids for miR-328, miR-214 and miR-1224-3p respectively (Figure 3B and 3C). Reporter assay revealed induction of miR-328, miR-214 and miR-1224-3p led to a marked decrease of luciferase activity of pGL3-WT-β-catenin-3′UTR plasmid, without change in luciferase activity of pGL3-MUT-β-catenin-3′UTR plasmid. These indicate that miR-328, miR-214 and miR-1224-3p directly modulate β-catenin expression by binding its 3′ UTR.

### EZH2 induces histone modifications and DNA methylation of miR-328

**Figure 4:**
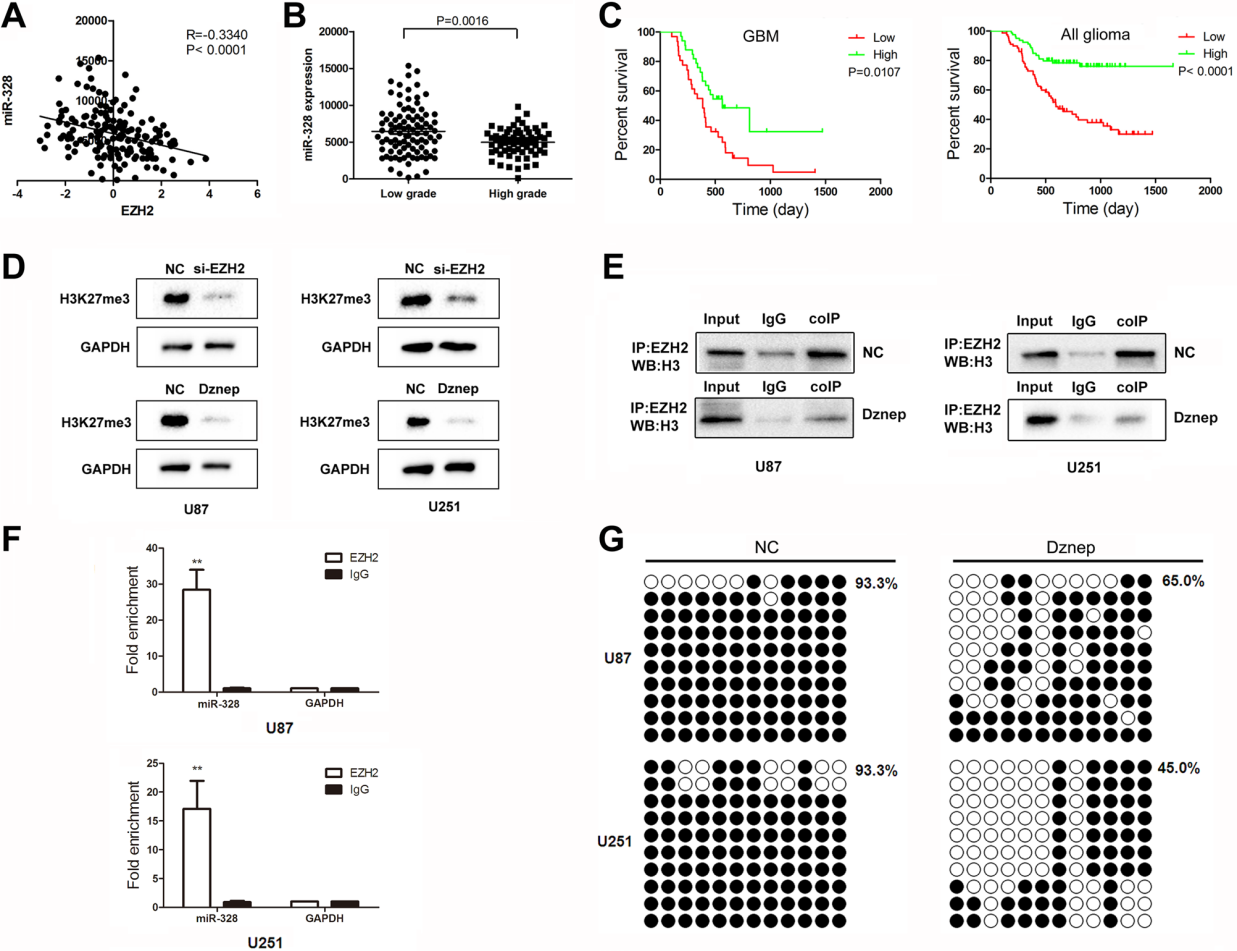
EZH2 induces histone modifications and DNA methylation of miR-328 (**A**) Correlation of EZH2 expression with miR-328 expression in 158 glioma tissues of CGGA data. (**B**) Level of miR-328 was analyzed in different glioma tissues of CGGA data. (**C**) Kaplan-Meier survival curves for miR-328 expression in GBM and 158 gliomas. (**D**) Western blot analysis of lysates from cells transfected by miR-328, probed with H3K27me3 antibody. GAPDH was served as the loading control. (**E**) Co-immunoprecipitation analyses of EZH2-H3 complex formation in U87 cells after Dznep treatment. (**F**) ChIP was performed on cell lysates using equal portions of anti-EZH2 as described in Materials and Methods. Input samples are DNAs amplified from lysates before immunoprecipitation. (**G**) Bisulfite sequencing of the miR-328 CpG island in glioma cells, Open and filled circles represent unmethylated and methylated CpG sites, respectively. Each horizontal row represents a single clone.

In order to explore the association of EZH2 with miR-1224-3p, miR-328 and miR-214, their expression profile of miRNA and gene arrays in 158 glioma tissues from CGGA data [9, 10] was analyzed. The Pearson correlation showed that a significant negative correlation of EZH2 expression with miR-328 and miR-1224-3p expression (*R* = −0.3340, *P* < 0.0001; *R* = −0.1647, *P* = 0.0355) (Figure 4A). And there were no direct correlation of EZH2 with miR-214 (*R* = 0.0046, *P* = 0.9536). Further, the level of miR-328 increased markedly in higher grade gliomas in comparison to lower grade gliomas (Figure 4B). As shown in Figure 4C, GBM samples expressing lower level of miR-328 were associated with decreased survival relative to those with lower level (*P* = 0.0107). And miR-328 expression was also inversely correlated with overall survival in 158 glioma patients.

EZH2, epigenetically silences many genes via the trimethylation of H3K27. As expected, Figure 4D showed that the level of H3K27 trimethylation was down-regulated in glioma cells by si-EZH2 and Dznep treatment. Also, immunoprecipitation experiments demonstrated that Dznep induced a significant dissociation of the protein interaction between EZH2 and H3 (Figure 4E). ChIP assays revealed that antibodies against EZH2 efficiently immunoprecipitated the miR-328 promoter regions (Figure 4F). Further, the degree of miR-328 promoter methylation was further demonstrated using BSP analysis. MiR-328 CpG islands were hyper-methylated at the methylation percent of 93.3% in U87 and U251 cells. Under the treatment of Dznep, the methylation degree decreased to 65.0~45.0% in glioma cells (Figure 4G). Thus, we suspected that miR-328 epigenetic silencing by EZH2 in gliomas was more complex involving both histone modifications and DNA methylation.

### MiR-328 is crucial for EZH2/β-catenin signaling in glucose metabolism

**Figure 5:**
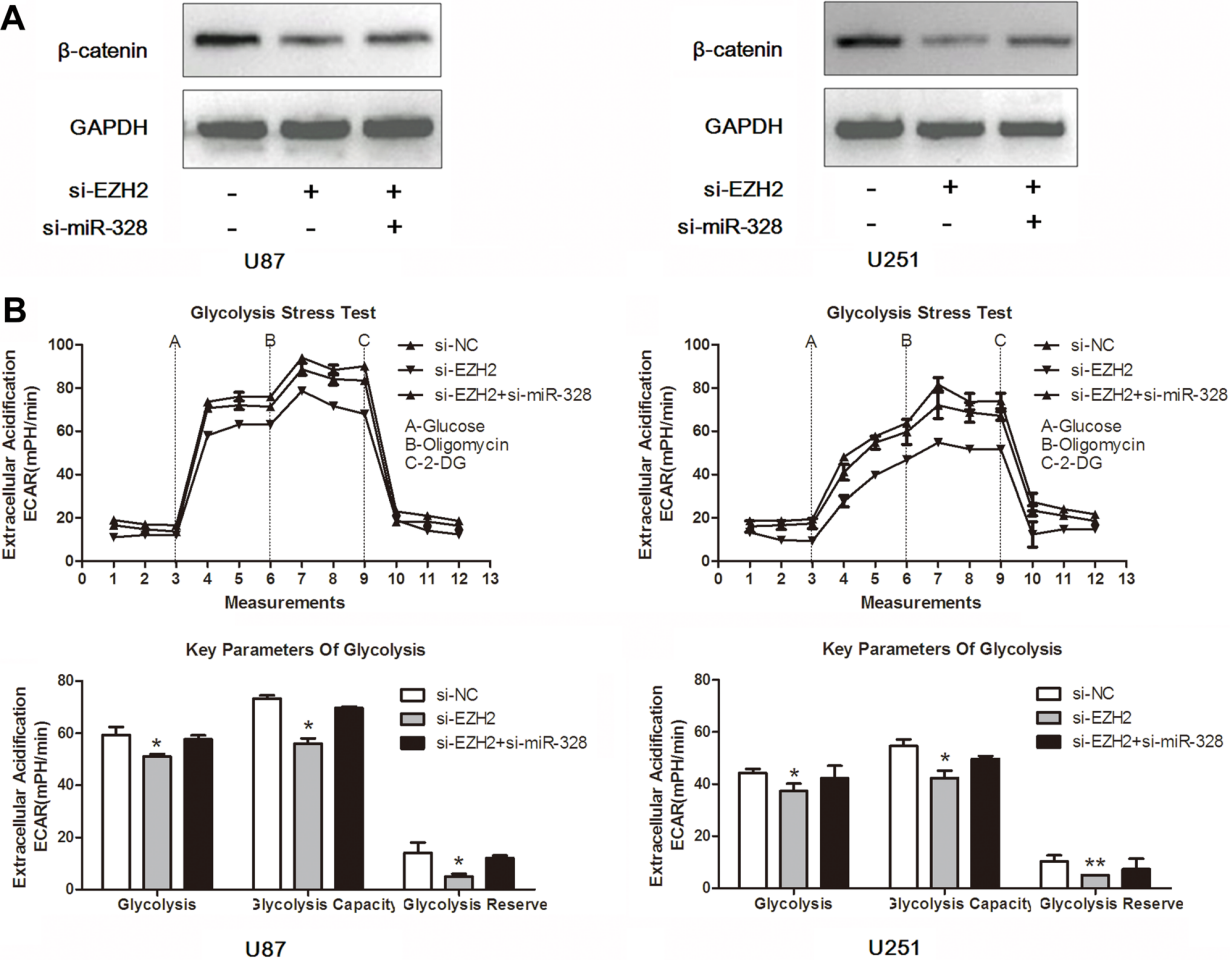
MiR-328 is crucial for EZH2/β-catenin signaling in glucose metabolism (**A**) Western blot analysis of lysates from cells transfected by si-EZH2 alone or in combination with si-miR-328 probed with β-catenin antibody. GAPDH was served as the loading control. (**B**) ECAR was measured by the Glycolysis Stress test in U87 and U251 glioma cell lines after the cells were treated with si-EZH2 alone or in combination with si-miR-328. *P < 0.05, **P < 0.01. Results are representative of at least three independent experiments.

Having demonstrated miR-328 as a direct target of EZH2, we next examined the importance of miR-328 in EZH2-mediated glucose metabolism. Co-transfection of si-EZH2 and si-miR-328 in glioma cells partially reversed β-catenin expression induced by si-EZH2 (Figure 5A). Moreover, si-miR-328 largely abrogated si-EZH2 effects on glucose metabolism in U87 and U251 glioma cells (Figure 5B). These results suggested that miR-328 was a critical mediator of EZH2/β-catenin signaling in glucose metabolism.

## DISCUSSION

Tumor cells exhibit high levels of glycolysis despite the presence of ample oxygen, a phenomenon termed the Warburg effect (aerobic glycolysis). Accumulating evidences suggest that targeting glucose metabolism may provide a selective mechanism by which to kill cancer cells. Several potential candidates that are overexpressed in certain cancer types include PKM2 [11], GLUT1 [12], HK2 [13], PHGDH [14] and LDH-A [15]. Here, we found that EZH2 was essential for glioma cell aerobic glycolysis. The inhibition of EZH2 activity by siRNA and Dznep decreased the level of the glycolysis under basal conditions, the glycolytic capacity and the glycolytic reserve.

EZH2 mediates histone methylation and recruits DNA methyltransferase in the silencing of a variety of genes, including miRNAs. In prostate cancer, several miRNAs that are repressed by EZH2 were identified, such as miR-181a, miR-181b, miR-200b, miR-200c and miR-203 [16]. In hepatocellular carcinoma, PRC2 complex epigenetically repressed miR-101 in a c-Myc-mediated manner, which in turn inhibited the expression of two subunits of PRC2 (EZH2 and EED), thus creating a double-negative feedback loop that regulates the process of carcinogenesis [17]. Here, we found several miRNAs repressed by EZH2, including miR-1224-3p, miR-328 and miR-214, regulate β-catenin expression by its 3′UTR in gliomas. Recent study showed that miR-214 negatively regulated EZH2 expression by targeting the EZH2 3′UTR [18]. It is possible that EZH2 and miR-214 establish a regulatory loop controlling PcG-dependent gene expression.

More studies have shown aberrant miR-328 in human malignancies including glioma [19, 20]. Previous data using miRNA expression profiles revealed that miR-328 showed reduced expression upon glioma progression [21]. Another research team also found miR-328 was significantly decreased both in anaplastic glioma and glioblastoma cohorts by miRNA arrays and real time PCR, and low miR-328 expression also conferred poor survival in primary glioblastoma patients [22]. Further ectopic miR-328 expression suppressed glioma cell proliferation. However, interestingly, Delic S reported that upregulated miR-328 promotes cell invasion in glioma cells [23]. In our study, miR-328 acted as a tumor suppressor by abrogating EZH2 effects on glucose metabolism in glioma cells.

In summary, we identified an EZH2/miRNA/ β-catenin feed-forward loop linking overexpression of EZH2, β-catenin and miRNA repression in glioma glucose metabolism. MiR-328 is a tumor suppressor miRNA and can be epigenetically targeted through EZH2 mediated histone modifications and DNA methylation. These findings represent a novel promising approach for silencing EZH2/miRNA/β-catenin amplification loop for combinatorial therapy of glioma.

## MATERIALS AND METHODS

### Cell culture and treatment

Human glioblastoma cells (U251 and U87) were obtained from the Chinese Academia Sinica cell repository (Shanghai, China). Cells were maintained in Dulbecco's modified Eagle's medium (DMEM, Gibco) supplemented with 10% fetal bovine serum, and incubated at 37°C with 5% CO2. Oligonucleotides were chemically synthesized and purified by high-performance liquid chromatography (GenePharma, Shanghai, China). The sequences are: EZH2 siRNA, 5′- GAGGGAAAGTGTATGATAATT −3′. EZH2 siRNA were transfected using Lipofectamine 2000 (Invitrogen). Cells transfected with nonsense siRNA oligonucleotides (scramble) were used as control. Dznep (Sigma) was purchased from Merck and was added with the final concentration of 5 μmol/l.

### Glycolysis stress test

The extracellular acidification rate (ECAR) was measured using the Seahorse XF96 Analyzer Glycolysis which calculates the net production and extrusion of protons into the extracellular medium. As glycolysis occurs, the resulting acidification of the medium surrounding the cells is measured directly by the XF Analyzer and reported as the ECAR. Initially, cells are incubated in glycolysis stress test medium without glucose. The ECAR refers to non-glycolytic acidification, which includes CO2 evolution followed by its hydration to carbonic acid and bicarbonate, as well as proton extrusion. The first injection is a saturating concentration of glucose. Glucose is taken up by the cells and catabolized to lactate, producing ATP and protons, with a corresponding rapid increase in ECAR. This glucoseinduced response is reported as the rate of glycolysis under basal conditions. The second injection is oligomycin. It inhibits mitochondrial ATP production and thus shifts the energy production to glycolysis, with the increase in ECAR revealing the maximum glycolytic capacity. The final injection is 2-DG, a glucose analog, which inhibits glycolysis through competitive binding to glucose hexokinase. The resulting decrease in ECAR further confirms that the ECAR produced in the experiment is due to glycolysis. The difference between the Glycolytic Capacity and Glycolysis Rate defines the Glycolytic Reserve.

### Human miRNA array

500 ng of total RNA from each sample was labeled and hybridized on Human miRNA OneArray according to the manufacturer's recommendations. Data was then average median normalized before generating differential expression values between treated and control samples.

### Real-time PCR

Real-time PCR was performed according to the manufacturer's instructions. The primers of miR-1224-3p, miR-328 and miR-214 were ordered from GenePharma Company. All experiments were performed using biological triplicates and experimental duplicates. The relative expression was calculated via the 2-ΔΔCt method.

### Western blot analysis

Equal amounts of protein per lane were separated by 8% SDS-polyacrylamide gel and transferred to PVDF membrane. The membrane was blocked in 5% skim milk for 1 h and then incubated with a specific antibody for 2 h. The antibodies used in this study were: EZH2, β-catenin, H3K27me3 (Cell Signaling Technology, USA). The antibody against GAPDH (Santa Cruz, USA) was used as a control. The specific protein was detected by using a SuperSignal protein detection kit (Pierce, USA). The band densities of specific proteins were quantified after normalization with the density of GAPDH.

### Luciferase reporter assay

The human β-catenin 3′UTR were amplified and cloned into the XbaI site of the pGL3-control vector (Promega, USA), downstream of the luciferase gene, to generate the plasmids pGL3-WT-β-catenin-3′UTR. pGL3-MUT-β-catenin-3′UTR plasmids were generated from pGL3-WT-β-catenin-3′UTR by deleting the binding site. For the luciferase reporter assay, cells were cultured in 96-well plates, transfected with the plasmids and miRNA mimics using Lipofectamine 2000. 48 h after transfection, luciferase activity was measured using the Luciferase Assay System (Promega).

### Methylation-specific PCR (MSP) and bisulfite sequencing PCR (BSP)

Genomic DNA from cell lines was isolated and subjected to bisulfite conversion and purification using EpiTect Fast DNA Bisulfite Kit (Qiagen). Then, MSP was performed by simultaneous use of primers for methylated and unmethylated forms. All PCR reactions were performed with negative and positive controls by using completely methylated and unmethylated human control DNA (Qiagen), respectively, as well as water. The BSP primers of miR-328 (forward)/ (reverse): (5′ - AGATTTATAGTATAGGGGGAGTTAGTGTGT −3′; 5′ - TCTAAAACAACCCAAAACTTCTCAC −3′). Amplified PCR Products were purified and cloned into pMD19-T (TaKaRa). 10 clones each cell were sequenced, respectively. Percentage of methylation was calculated comprehensively and comparatively by Biq-analyzer.

### Chromatin immunoprecipitation

The assays for chromatin immunoprecipitation (ChIP) were performed using reagents commercially obtained from Upstate Biotechnology and conducted essentially according to the manufacturer's instructions. Briefly, cells were maintained in 100 mm cell culture plates and were then fixed with formaldehyde for 10 min. Cells were lysed in SDS lysis buffer, and the chromatin DNA was extracted and sonicated into 200–1000 bp fragments. Purified DNA was used for PCR amplification. The primers of miR-328 (forward)/ (reverse): 5′ - ACCCAAGTTGCGAATGTGAG −3′; 5′ - TCCAGCCGTAGGTTGTGAAT −3′. Each target site was calculated as 2 to the power of the cycle threshold difference between input DNA and ChIP samples. Enrichments at target sites were compared with negative/unbound control region GAPDH.

### Nude mouse glioma intracranial model

U87 cells were transfected with lentivirus overexpressing si-EZH2 and ontaining a luciferase reporter *in vitro* for 2 days. A total of 2 × 10^5^ U87 cells infected with virus were implanted stereotactically to establish intracranial gliomas using cranial guide screws. Mice were imaged for Fluc activity using bioluminescence imaging (BLI) on day 7, 14 and 21.

### Immunoprecipitation

Cells were collected and lysed using lysis buffer supplemented with PMSF. Then, equal amounts of protein was subjected to anti-EZH2 antibody (CST, USA) following overnight incubation at 4°C. Following this, protein-antibody immunoprecipitates were collected by protein A/G plus-agarose (Santa Cruz, USA). Finally, the EZH2 and H3 protein was analyzed by Western blotting.

### Statistical analysis

A t-test was used to analyze differences in each two-group comparison, while one-way ANOVA was used to determine the difference among at least three groups. Kaplan-Meier analysis was employed to assess the survival rate of patients. *P* < 0.05 was considered to be a statistically significant difference.
